# Capturing of intracranial pressure treatment during neurointensive care in patients with acute brain injury using a novel tablet-based method

**DOI:** 10.1007/s10877-022-00820-6

**Published:** 2022-02-01

**Authors:** Peter Galos, Lena Nyholm, Tim Howells, Per Enblad

**Affiliations:** 1 Department of Neuroscience/Neurosurgery, Uppsala University, Uppsala, Sweden; 2 Department of Surgical Sciences/Anesthesia and Intensive Care, Uppsala University, Uppsala, Sweden

**Keywords:** Critical care, Manual measures, Registration system, Intracranial pressure, Treatment

## Abstract

Critical care is complex and stressful. It is difficult to register in real time data not recorded by automatic systems. Time-specific knowledge of *manual measures* is important for understanding pathophysiology and for analyzing treatment and quality of care. Therefore, a novel iPad-based method for registration of manual measures was developed, which many can build themselves. Using a configuration for intracranial pressure (ICP) management, the methodology was validated, ICP treatment captured, and the quality of ICP management evaluated. Twenty-two patients with acute brain injuries were studied. The iPad-system was totally used for 2538 h. Thirteen-hundred-five manual measures were entered. Thirty-nine episodes of predefined ICP insults were identified. During 16/39 episodes, ICP treatments were registered. For 4/39 episodes treatments were registered within 90 s before or after the episode. For 3/39 episodes it was registered that treatment was intentionally refrained. In 15/16 episodes without registered treatment, the insult was mild or reasonable explanations were found when medical records and the Patient data management system were reviewed. In one situation without particular circumstances, morphine and clonidine were given to decrease ICP but not registered. No episodes of downtime or loss of data occurred. The developed methodology appears to be stable and robust as well as feasible and user-friendly. It was possible to capture the treatment of ICP insults with high temporal resolution, and to evaluate the quality of ICP management. An own developed novel tablet-based system like our system may be a promising potential tool useful in various future intensive care applications.

## Introduction

The focus on avoiding secondary insults, e.g. raised intracranial pressure (ICP), hypotension or fever, in neurointensive care (NIC) of traumatic brain injury (TBI) has improved outcome substantially [1, 2]. Multimodality monitoring makes it possible to detect insults at an early stage, so that treatment can be initiated before secondary brain injury becomes manifest. The secondary insult concept has been generalized to all types of acute brain injuries. Standardized care [3] and computerized collection of monitoring data makes the NIC unit to an excellent research platform which also may be used for quality assurance of care [4]. The work in a NIC unit is complex and at the same time often stressful. Data not registered by automatic systems, e.g. administration of certain drugs or change in the body position of the patient, may therefore not be registered properly. Time specific knowledge of these so-called *manual measures* may be an important piece of the puzzle in understanding pathophysiological processes and in determining the cause-effect relationship of clinical decisions as well as for monitoring the compliance to treatment protocols and development of AI systems for intensive care.

There have been attempts to map the NIC treatment using observers with pen and paper, and bed-side desktop computers, respectively, to register manual measures [5]. However, besides from being time- and resource consuming, analysis of data from the studies indicates that the time precision of the registrations to a large extent are imprecise. This is probably due to the demanding environment in combination with the complicated registration processes where registrations of treatment often appear to be done in retrospect, e.g. at the end of a nursing shift. An integrated patient data management system (PDMS) offers the possibility to register manual measures, but in reality, there are many challenges, e.g. difficulty to get a user interface tailored for the specific clinical purpose or research project due to limitations regarding configurations and screen options, centralized hospital governing, required company involvement and demanding login and multi-click procedures. In recent years the development of consumer tablets, such as Apple iPad, provides the potential to build yourself with minor programming experience, at site in the intensive care unit, a userfriendly, affordable and reliable digital system for more efficient and correct registration of manual measures customized for specific purposes in the unit.

The *objectives* of this pilot NIC study were to: (1) Describe the development, by one of the authors (P.G.), of our own novel tablet-based method for manual registration of treatment actions beyond the scope of existing digital systems. (2) Validate the methodology using a configuration for ICP management. (3) Capture the treatment of ICP insults by using our novel iPad-based method. (4) Evaluate the quality of ICP management.

## Methods

### Patients

Patients older than 15 years, with ICP monitoring, treated at the NIC for traumatic brain injury, subarachnoid haemorrhage or intracerebral hematoma were eligible for the study and conveniently included.

### Neurointensive care

The NIC unit at Uppsala university hospital receives patients from the Uppsala region and the neighbouring regions with a total population of 2 million inhabitants. The NIC follows standardized protocols emphasizing that prevention and intensive treatment of secondary insults are of outmost importance to avoid secondary brain injury [3]. All patients not responding to commands (Glasgow coma motor score 1–5) are mechanically ventilated and have ICP monitoring. Mechanically ventilated patients receive propofol for sedation and morphine as analgesia. For ICP monitoring, a ventricular drainage catheter is used as first choice or alternatively an intraparenchymal sensor if the ventricular system is compressed. ICP above 20 mmHg should be treated in an escalated manner by using different options depending on type of brain injury, e.g., increased sedation, hyperventilation, cerebrospinal fluid (CSF) drainage, barbiturates and decompressive craniectomy.

### General system development

Several requirements were itemised for our manual recording system. The user interface should be intuitive and self-explanatory for ICU staff. Units should always be turned on, be in close reach bedside and be operated with a one-click procedure. The system should fulfil hygiene, IT Security and Electrical safety standards, and be approved by the hospital Medical Technology Department for ward deployment. The system should be reliable in terms of stability, affordable, modifiable and scalable for use in other studies. Bluetooth and Wi-Fi interfaces should be provided for access to other systems.

The system, which we named *Capture Intensive Care* (CIC), was developed to meet those requirements. It is based on Apple iPad and the software was programmed in Objective C using Apples XCode [6] environment. Data recorded by ICU staff is encrypted and sent from the iPad through a SOAP/WSDL service to a server programmed in PHP/HTML where it is stored in a SQL database. The system can be configured by the user. It is scalable and can collect and merge data, from a more or less unlimited number of units simultaneously, to a single database. The system can be reconfigured for other studies, using a different user interface and has the possibility to read data from bedside instruments or systems through Bluetooth, Wi-Fi or cables. Data is registered by a one-click procedure. CIC can be integrated and deliver data to different analysis tools, for research purposes or in real time clinical decision making. The total cost per unit is 400 USD and the approximated life span > 5 years.

The registration starts when the iPad is configured with the patient’s study ID. All registrations, consisting of patient ID, timestamps and types of intervention, are stored in the iPad. Once per minute, a synchronization over Wi-Fi of encrypted data to a server is performed automatically, and the local copy is erased upon confirmation from the server.

### Configuration for registration of ICP interventions and implementation

**Fig. 1:**
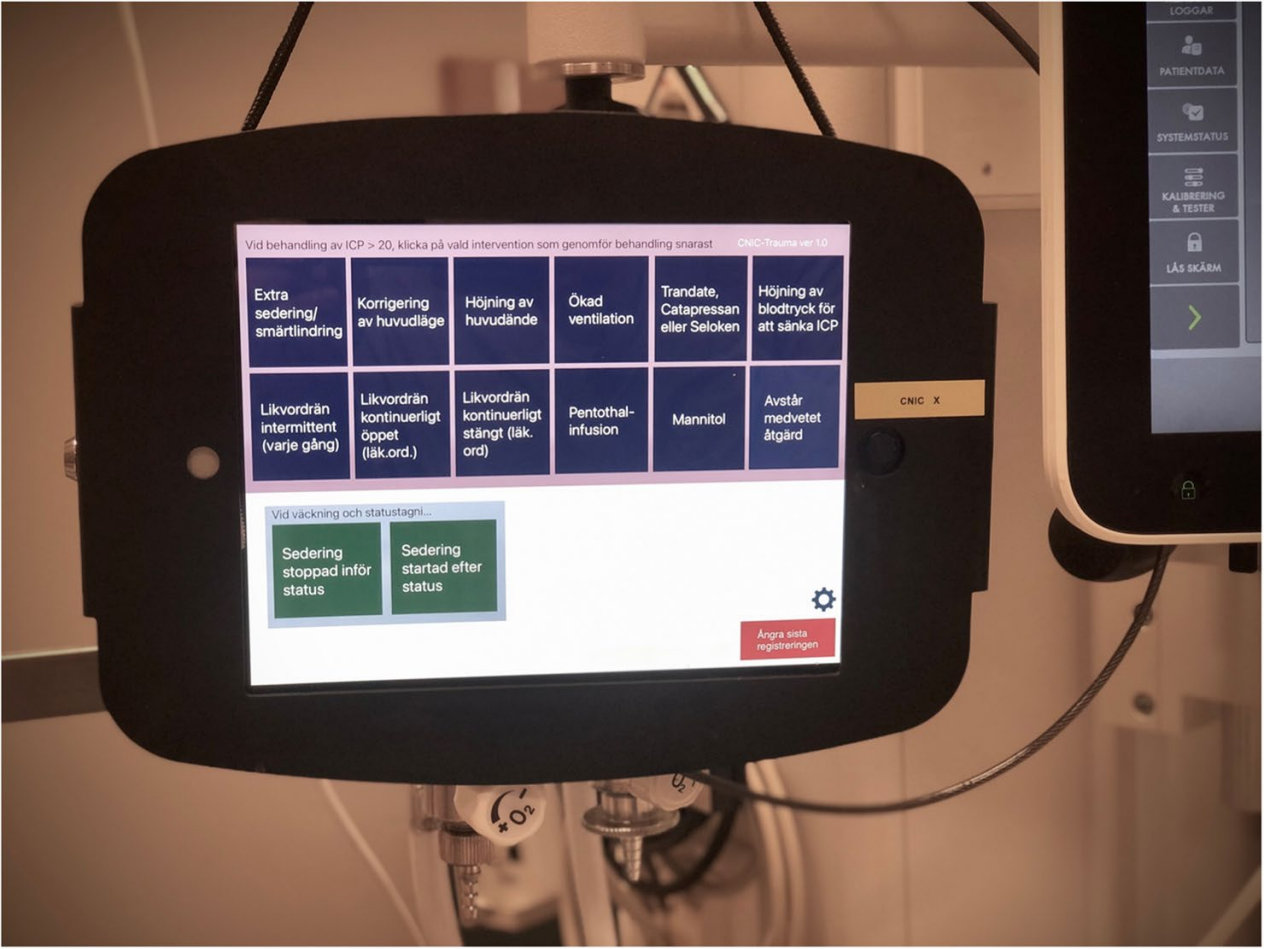
CIC configured for the ICP study bedside at NIC. Camera and microphone were blocked by duct tape. The iPads were encapsuled in steel casings and locked to the wall using steel wires and code padlocks. The iPads were cleaned using same procedure as for medical equipment, i.e., carefully wiped with surface disinfectants, without any loss of functionality

To analyse treatments aiming to decrease a raised ICP, CIC was configured to register ICU staff entries of manual measures. After a few pilot versions and minor modifications, a final study configuration was accomplished (Fig. 1). An *entry* is the data collected when a button is pressed on CIC. Entries include *registrations* and *clinical notations.* The registrations denote to different treatments of increased ICP (Table 1). The clinical notations were pre-configured and applied in this study to a procedure not associated to acute ICP treatment, i.e. “Start of sedation” and “End of sedation” due to wake-up tests or a request to “Exclude the last entry” (Table 1). Before initiation of the study, the CIC system was demonstrated for the critical care nurses both at lectures and at bed-side.

**Table 1:** Entries divided in registrations and clinical notations

Entries	Collected
Registrations	
Extra anaesthesia/analgesia	382
Correction of head position	105
Increase in head-of-bed elevation	85
Intermittent opening of ventricular drain	320
Continuous opening of ventricular drain	51
Increased ventilation (respiratory minute volume)	12
Anti-hypertensive treatment: labetalol, clonidine or metoprolol	4
Thiopental sodium infusion	2
Mannitol infusion	2
Increase of blood pressure to reduce ICP	40
Treatments total	1003
ICP high, intentional refrained treatment	302
Registrations total	1305
Clinical notations	
End of sedation due to wake up test	90
Start of sedation after wake up test	71
Start of new patient registration	22
Exclude the last entry	99
Clinical notations total	282
Overall total	1587

### Data processing and analysis of data

For the validation of CIC and the evaluation of ICP management, the CIC entries were related to automatically collected ICP data. High frequency physiological patient monitoring data are routinely collected from the bedside monitors, by the ODIN data monitoring and collection system, developed in Edinburgh and Uppsala by Tim Howells and colleagues [7]. Invasive physiological data, e.g. arterial blood pressure and ICP, is stored in the ODIN database. The clocks of CIC and ODIN systems are both synchronized with a time service to avoid discrepancies. Collected registrations from CIC were merged with 1 Hz (the 1 s mean value) ODIN data of intracranial pressure to combine the time-stamped treatment information from CIC with the collected physiological monitoring data. The study period was 3 days or longer if there were ongoing ICP problems.

**Fig. 2:**
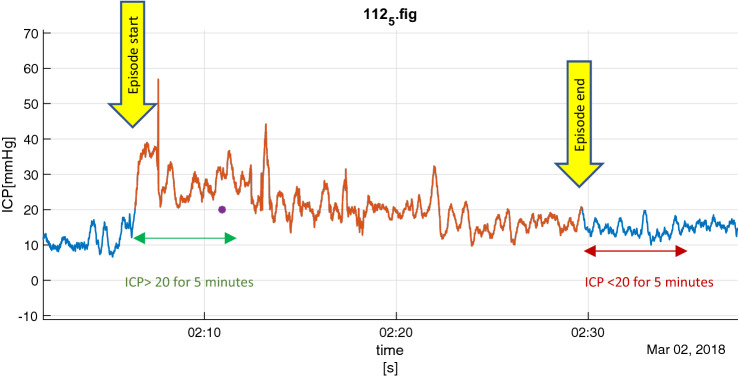
Definition of a potential ICP insult with start and end of episode marked with yellow arrows

The start of a potential ICP insult was defined as when ICP is above 20 mmHg for 5 min. The timespan of the ICP insult stretches from the start of such episode until the start of when ICP is below 20 mmHg for 5 min (Fig. 2). The NIC staff were not informed about the definition and were expected to treat according to the management protocol, i.e. when ICP was above 20 mmHg for a few minutes.

### Statistics

The study was mainly descriptive.

## Results

Twenty-two patients were included in the study. There were 14 men and 8 women with a mean age of 51 years (range 19–73). Nine patients had traumatic brain injury, 7 aneurysmal subarachnoid haemorrhage and 6 intracerebral hematoma. The CIC system was totally used for 2538 h. No episodes of downtime or loss of data appeared for the CIC system during the study period.

There were 1587 CIC entries; 1003 treatments, 302 decisions to refrain treatment and 282 clinical notations. The most common treatments to reduce a raised ICP were 382 occasions of extra anaesthesia/analgesia, 320 intermittent openings of ventricular drain and 190 changes in elevation (85) or position (105) of the head (Table 1).

**Fig. 3:**
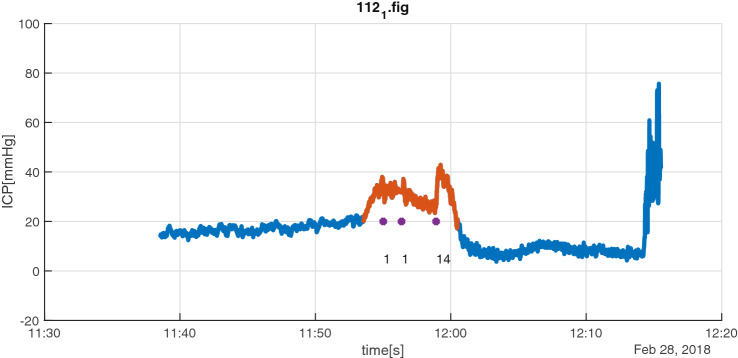
Group A insult episode (red) with three registered interventions (purple markers) inside timespan: initially two attempts of increased anesthesia/analgesia (1) followed by an induced increase in blood pressure (14) to decrease ICP

**Fig. 4:**
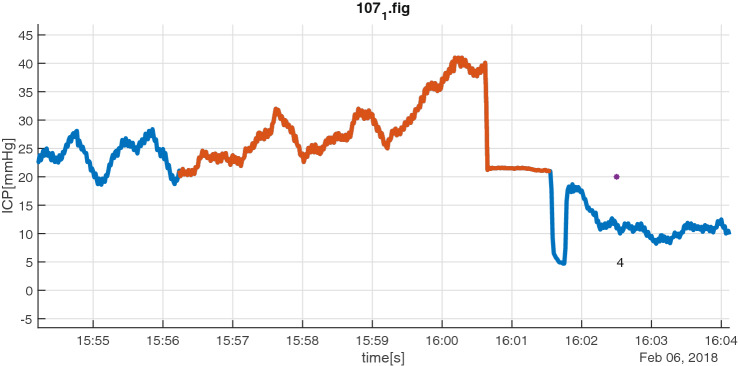
Group B insult episode (red) with registrations ± 90 s outside interval: in this case registration of opening of ventricular drain (purple marker/4) 60 s after end of episode. The actual time of opening is probably 2 min before the registration due the rapid decrease in ICP at that point of time

**Fig. 5:**
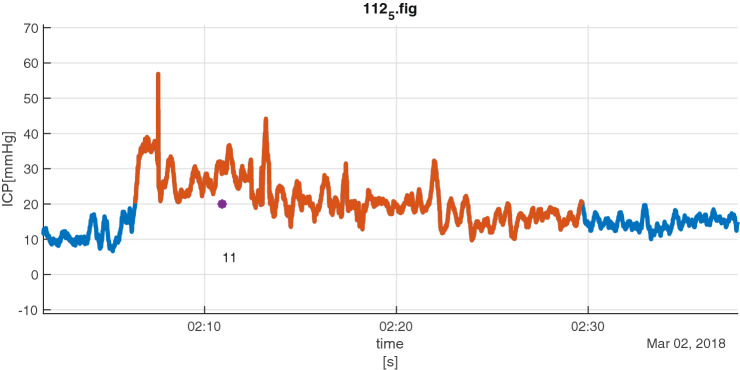
Group C episode (red) where the critical care nurses registered that they intentionally refrained from treatment. Purple marker indicates when the decision was registered

When the captured ICP treatments were analysed in relations to collected ICP data, 245 of the 1587 CIC entries had to be excluded, due to missing monitoring data during transportation to surgery, radiology, etc. Applying our definition of an ICP insult on collected monitoring data from ODIN, 39 episodes were identified. Four patterns could be discerned when the identified episodes of potential ICP insults were related to registrations in CIC. In 16/39 episodes (41%), one (5/16) or more (11/16) treatment of the raised ICP was registered during the defined insult timespan (*Group A*, see Fig. 3 for example). In 4/39 episodes (10%) there were no treatments registered during the insult timespan but within 90 s before start or within 90 s after the end of the defined period (*Group B*, see Fig. 4 for example). For 3/39 (8%) episodes the staff had registered that they noted a high ICP but intentionally refrained from (all) treatment for the entire episode (*Group C*, see Fig. 5 for example). There were 16/39 episodes (41%) without registered interventions and notations of intentionally refrained treatment (*Group D*).

**Fig. 6:**
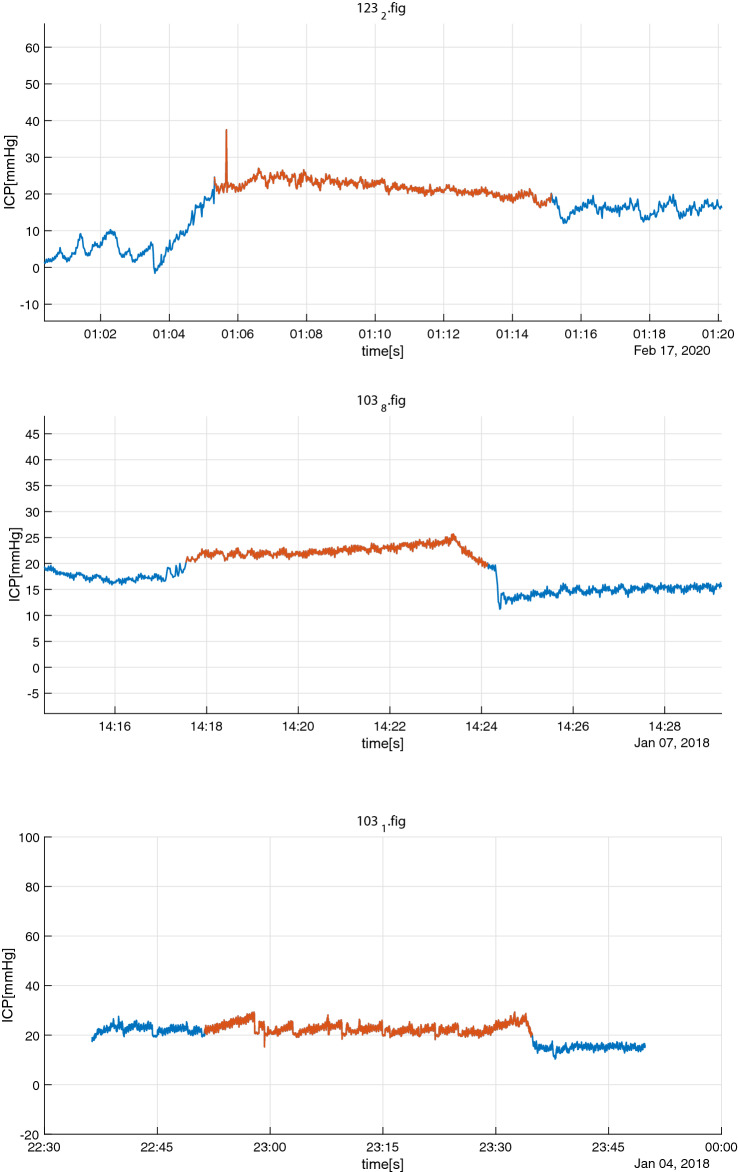
Group D episodes (red) fulfilling our definition of ICP insult without registered treatment which may be explained by that ICP was only slightly increased

In 3 of the 16 cases (*Group D*) without registered ICP treatment, the episode could be classified as mild with ICP only slightly elevated above 20 mmHg during the insult period (see Fig. 6). Using medical records and notes from the Patient data management system, reasonable explanations why there was no registered intervention were found in 12 of the remaining 13 Group D episodes: In 5 episodes the ICP insult corresponded to neurological examination/pain stimulus (3 with ongoing sedation and 2 where sedation was restarted right after the end of episode). Two episodes were associated with extubating procedures and another 3 were explained by respiratory problems with simultaneous hypoxia, hypertension, tachycardia and/or notification of upper airway suction. During one episode, there was an occlusion in the ventricular drain which had to be flushed by the neurosurgeon on call. In one of the situations thiopental was repeatedly administered during transportation to acute CT. In another situation, morphine and clonidine were given to decrease ICP but not registered in CIC.

In some of the insult episodes multiple interventions were registered, simultaneous or consecutive, as treatment of the raised ICP. The strategy of intervention differed, but a common method was repeated doses of sedation, sometimes in combination with a change in head position and/or opening of the CSF drain.

The clinical notation “Stop of sedation for wake-up test” with corresponding complete ODIN monitoring data was found 71 times. In 44/71 (57%) of the registrations, a matching “Start of sedation after wake-up test” was also registered. The mean time interval from stop to (re)start of sedation was 36 min (range 1–89). In the 27/71 (38%) cases a registration of start of sedation (after wake-up test) was missing.

## Discussion

### Validation of the developed tablet-based method for capturing treatment actions

The CIC system appeared to be stable and robust since no episodes of downtime or loss of data appeared during the study including a significant number of patients and substantial amount of monitoring time. The finding that the staff had registered either a treatment of ICP or a decision to refrain treatment within or close to 23 of the 39 (59%) identified insult episodes indicates that the CIC system is feasible and user-friendly in the intensive care environment. This is further supported by that reasonable explanation for missing registrations were found in all but one of the remaining 16 insult episodes when additional information from the medical records and the PDMS were reviewed.

One challenge using the CIC system is that the novelty use by the bedside nurses may wane. The best way of using the system is probably not continuous use. Our believe is that the system is more suitable for intermittent use during restricted periods with changing purposes and tailored configurations for the specific aims. It is of outmost importance that the nurses receive information about the purpose of a new project and have some bed-side training before it starts.

### Captured ICP treatment and quality of care

Analysis of the treatments registered in the CIC system showed that as much as 1003 actions had been taken to lower ICP, and that extra anaesthesia/analgesia and intermittent opening of the ventricular drain was the far most common measures. Looking at the 39 identified ICP insults, it was obvious that treatment was given or there were reasonable explanations for refraining treatment in virtually all events. In 302 situation the critical care nurses had notified that they intentionally refrained treatment but monitoring data showed that only in 3 of those situations the episode fulfilled the predefined criteria for a potentially harmful ICP insult. Information about the reasons for deciding to refrain from ICP treatment is not available, but the most obvious reason was probably that the ICP elevation was judged to be mild since only 3 out of the 302 notations were associated with an identified episode of elevated ICP.

Many observations indicate that quality of ICP management was good. First, a large number of ICP treatments were given. Second, only 39 ICP insult episodes were identified according to the predefined criteria during 2538 h of ICP monitoring. Third, a large proportion of the identified ICP insults were actively treated or there was an active decision not to treat when the insult was judged to be mild.

Thirteen percent of the identified ICP insults were connected to airway management and extubating procedures. This illustrates the importance of carefully managing the extubating procedure in terms om timing and preparing for possible adverse events. An algorithm, including neurological and respiratory function as well as current ICP instability, could be valuable in the decision process when to extubate.

### Limitations

The mixture of different types of brain injuries and the relatively small number of each diagnosis limits the possibility to analyze the treatment measures in more detail and in relation to the management protocols for the specific brain injuries. However, the present study should be looked upon as a pilot study aimed at validating the tablet-based CIC system and at getting proof of the concept that the CIC system may be used to capture intensive care treatment. Regarding the potential usefulness of the CIC system for quality of care evaluation (in this study quality of ICP management), a study-bias cannot be excluded and therefore additional complementary blinded methods needs to be applied for quality assurance, e.g. monitoring of the amount of ICP insults.

## Conclusions

Our novel CIC system appears to be stable and robust as well as feasible and user-friendly in the intensive care environment. It was possible to capture the treatment of ICP insults during NIC with high resolution of time and to evaluate the quality of care in this pilot study. Thus, own developed novel tablet-based systems like our CIC system may be promising potential tools useful in various future intensive care applications when correct time stamps are required.
